# Supplementary material for: Phage display demonstrates durable differences in serological profile by route of inoculation in primary infections of non-human primates with Dengue Virus 1
Source: Sci Rep. 2021 May 24;11:10823. doi: 10.1038/s41598-021-90318-z (PMC8144558; doi:10.1038/s41598-021-90318-z)
Supplement: Supplementary file 1 — Supplementary Information. [file 41598_2021_90318_MOESM1_ESM.docx]

**Supplemental Data**

Phage display demonstrates durable differences in serological profile by route of inoculation in primary infections of non-human primates with Dengue Virus 1

Jayant V. Rajan^*1^, Michael McCracken^2^, Caleigh Mandel-Brehm^1^, Greg Gromowski^2^, Simon Pollett^2^, Richard Jarman^2^, Joseph L. DeRisi^1,3^

^1^University of California, San Francisco, San Francisco, CA, USA

^2^Walter Reed Army Institute of Research, Silver Spring, MD, USA

^3^Chan-Zuckerberg Biohub, San Francisco, CA, USA

^*^Corresponding Author (email: Jayant.Rajan@ucsf.edu)

**
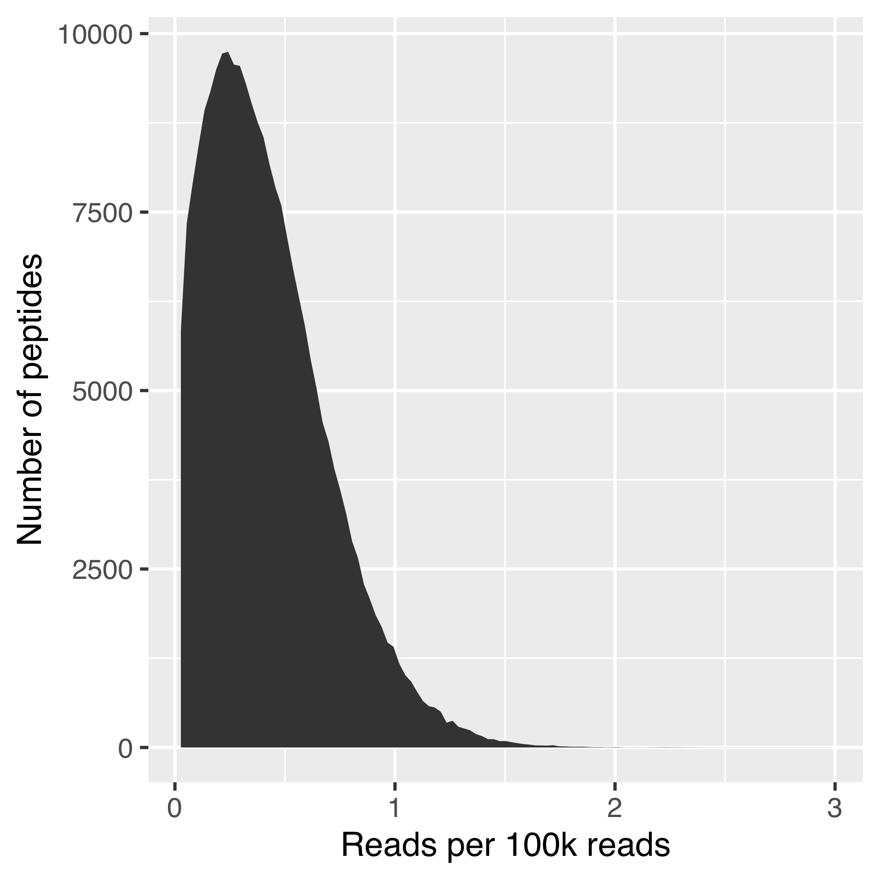
**

**Supplemental Figure 1. Peptide representation of packaged phage display library**. Library oligonucleotides were amplified and cloned into the T7 phage display vector as described in Methods. At a sequencing depth of 10.7 million reads, 96.7% of designed peptides were represented. Of the peptides represented, 96% were present at less than or equal to 1 read per 100,000 reads.

**Supplemental Figure 2. Input library abundance does not bias selection of significant peptides.** Reads per 100,000 reads in the input sequenced library and after PhIP-Seq are shown for peptides called as significantly elevated over baseline across all samples spanning all 20 nonhuman primates included in this study. The most significantly elevated peptides are not skewed towards the most abundant peptides in the sequenced input library.

**Supplemental Figure 3. Multiple sequence alignment of differentially expressed region in the dengue virus envelope protein domain III.** A multiple sequence alignment of reference sequences of the envelope (E) protein for each of the dengue virus (DENV) serotypes was done. The residues corresponding to the region that was differentially expressed at late timepoints in mosquito-infected non-human primates versus subcutaneous-injection infected primates is shown. Across the length of the region, 16/46 positions (34.7%) were 100% conserved across all four DEVN serotypes.

**Supplemental Figure 4. Multiple commercially available flavivirus antibodies enrich for their expected target when it is displayed on bacteriophage T7.** Immunoprecipitations were performed with commercially available antibodies to the flavivirus fusion loop (4G2), DENV2 NS3, and WNV M protein. A total of 2 rounds of phage PhIP-Seq immunoprecipitations were performed for each antibody. In each case, there was enrichment for the target sequence as compared to the abundance of peptides containing the target sequence of each antibody in the sequenced input library used for the PhIP-Seq experiments.


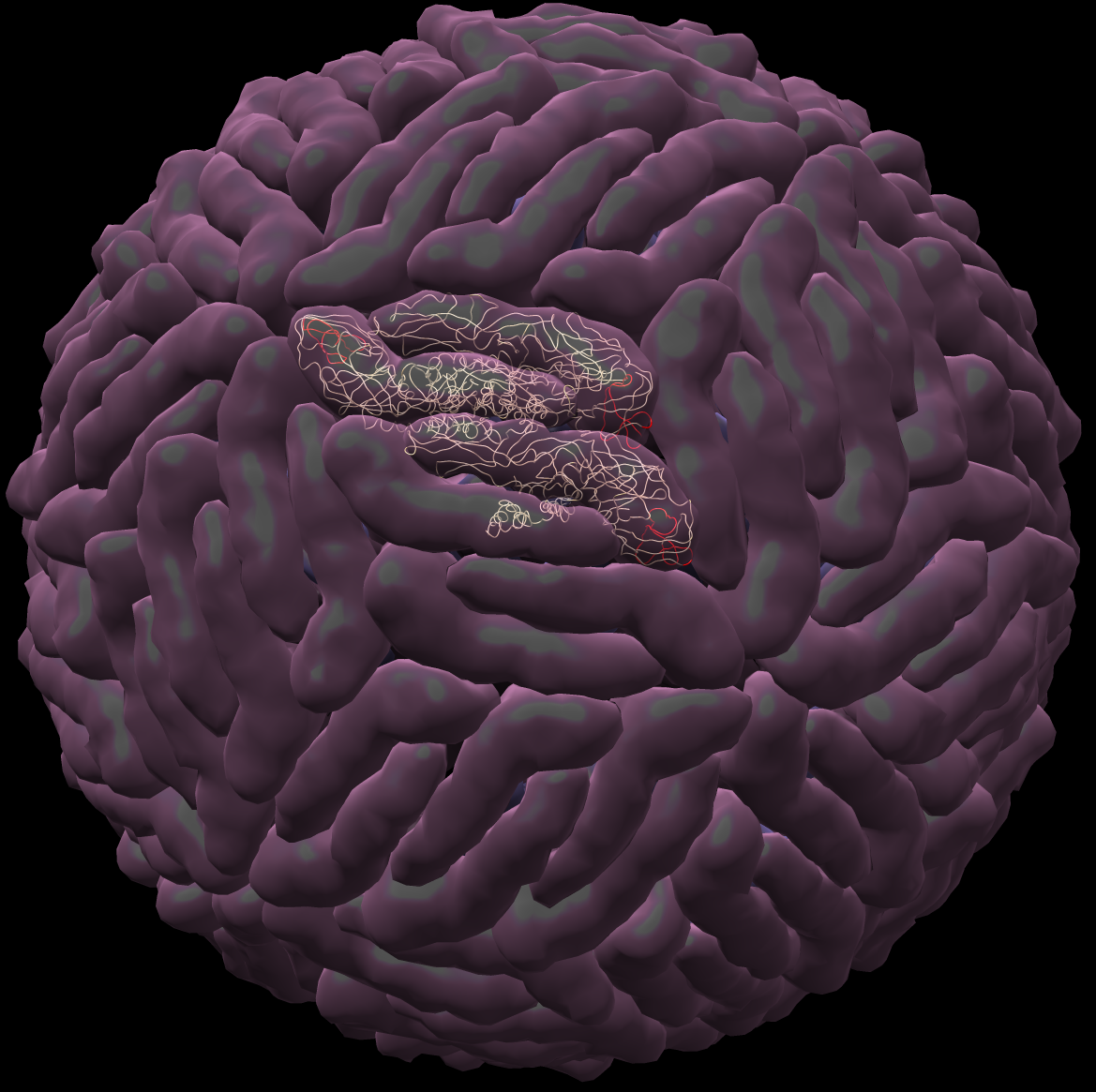


**Supplemental Figure 5. Dengue 1 virion with highlight of differentially expressed envelope protein domain III region.** A structure of the DENV1 envelope protein was obtained from [www.pdb.org](http://www.pdb.org) (4cct.pdb) and a model of the virion generated using UCSF Chimera software. Three envelope protein monomers are shown with the region in domain III of the envelope protein which showed higher seroreactivity in mosquito-inoculated versus subcutaneously inoculated animals highlighted in red.


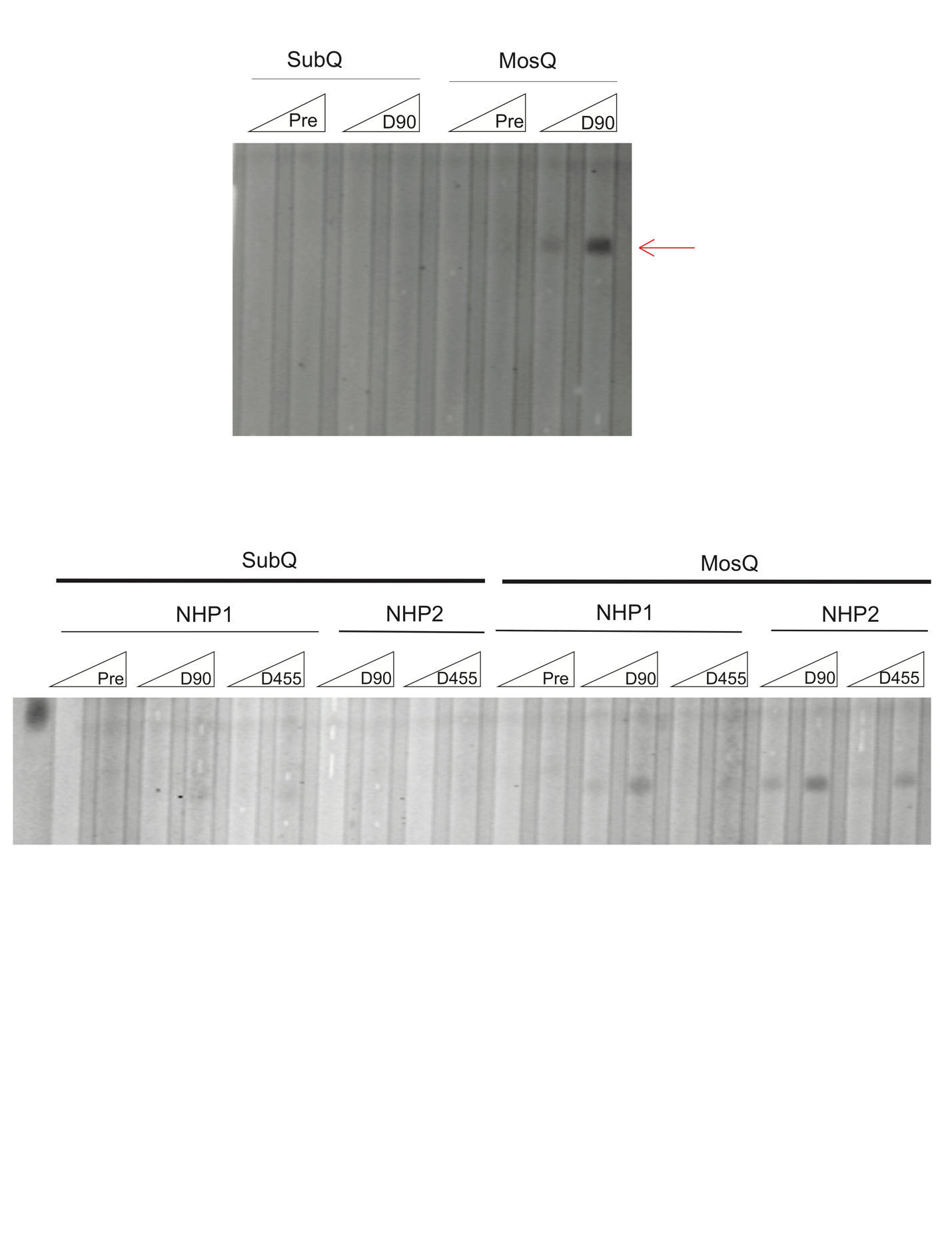


**Supplemental Figure 6. Persistent antibodies against envelope protein residues 339-384 in macaques inoculated by mosquito.** Slot immunoblots were performed using sera from macaques inoculated by mosquito bite and by subcutaneous injection. Results in 1 pair of animals showed that the signal was absent at baseline in both groups but present at 90 days post-infection, with the signal titrating (A). The same results were obtained in two other animals from each group, extending to 455 days post-infection (B). These results were consistent with PhIP-Seq results. All Sera were diluted 1:100 in PBS for immunoblots.

B

A


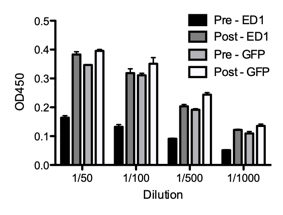

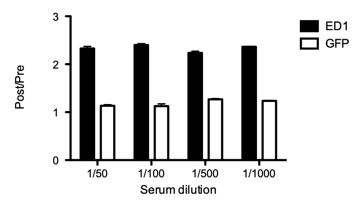


A

A

C


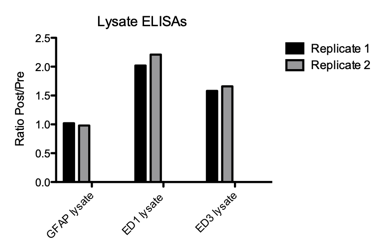


**Supplemental Figure 7. Titrations of ELISAs for two different envelope protein domains that are enriched by PhIP-Seq.** Constructs spanning envelope protein residues 1-91 and 339-384 were cloned into a mammalian expression vector and overexpressed in HEK293T cells. A FLAG tag was added to the carboxy terminus of each construct. Whole cell lysates were captured using an anti-FLAG antibody (Cell Signaling, Inc), diluted macaque sera added and detected with a an anti-monkey IgG HRP conjugated antibody (Sigma, Inc). Signal, as measured by OD450 for both constructs titrated with decreasing amounts of serum (A). The same was true for OD450 ratios of post-exposure (90 days) to pre-exposure (B). Ratios for a control construct (GFP) remained ~1.0, while those for a construct against ED1 and ED3 increased 90 days post-exposure (C).


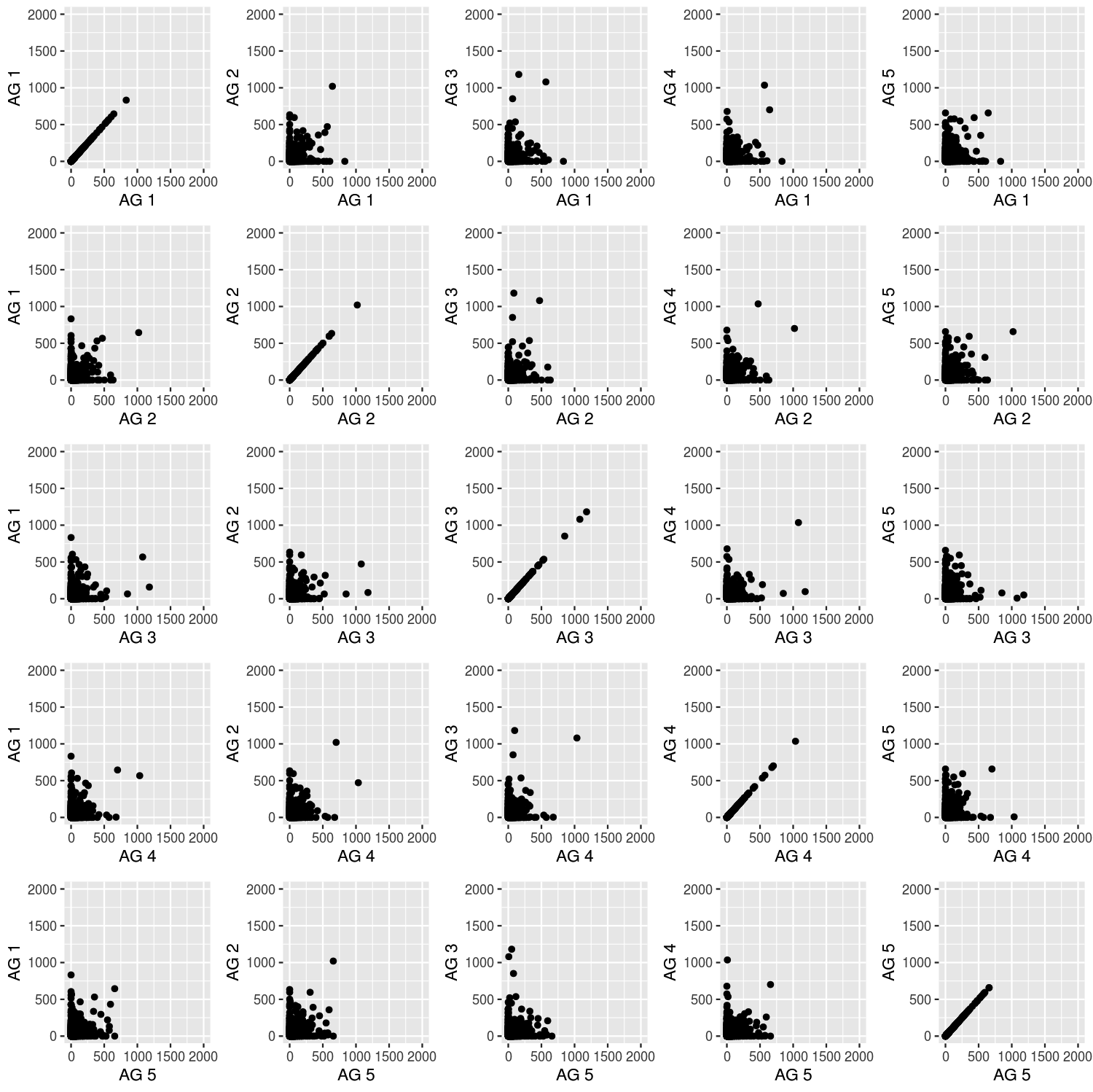


**Supplemental Figure 8. Correlations of no-serum bead controls for PhIP-Seq.** Mock immunoprecipitations with no serum present were done using our flavivirus phage display library. Independent beads-only mock precipitations were not highly correlated with eachother, consistent with background binding in the absence of serum being largely stochastic.

**Supplemental Figure 9. Correlations between replicates of PhIP-Seq experiments.** Replicate immunoprecipitations were performed for a randomly selected subset of samples and reads/100k reads calculated for each sample. Pairwise correlations between all samples were determined by calculating Pearson correlation coefficients. Replicates of the same sample were highly correlated. Some non-replicate pairs were also correlated, in each case reflecting longitudinal samples from the same individual.

| **Animal** | **Gender** | **Weight** | **Age** |
| --- | --- | --- | --- |
| Subcutaneous 1 | Female | 4.52 | 5.53 |
| Subcutaneous 2 | Female | 5.26 | 4.64 |
| Subcutaneous 3 | Female | 5.08 | 4.64 |
| Subcutaneous 4 | Female | 4.42 | 4.61 |
| Subcutaneous 5 | Female | 5.82 | 4.61 |
| Subcutaneous 6 | Female | 4.18 | 4.61 |
| Subcutaneous 7 | Female | 4.04 | 4.60 |
| Subcutaneous 8 | Female | 5.06 | 4.59 |
| Subcutaneous 9 | Female | 4.62 | 4.57 |
| Subcutaneous 10 | Female | 5.54 | 4.56 |
| Mosquito 1 | Female | 5.76 | 4.37 |
| Mosquito 2 | Female | 5.08 | 4.41 |
| Mosquito 3 | Female | 5.64 | 4.45 |
| Mosquito 4 | Female | 5.28 | 4.46 |
| Mosquito 5 | Female | 4.78 | 5.53 |
| Mosquito 6 | Female | 4.92 | 5.54 |
| Mosquito 7 | Female | 3.98 | 9.63 |
| Mosquito 8 | Female | 5.24 | 5.67 |
| Mosquito 9 | Female | 5.22 | 5.60 |
| Mosquito 10 | Female | 5.48 | 5.57 |

**Supplemental Table 1. Characteristics of non-human primates.** Shown in the table are the genders, weights and ages of each of the animals (at baseline) that were included in this study. There was no significant difference in the mean weights (p=0.26) or ages (p=0.13) of animals between the two groups, despite the presence of a single outlier animal (Mosquito 7) in one of the groups.
